# Shall Midwives Be Licensed?

**Published:** 1885-04

**Authors:** 


### Editorial.

### Shall Midwives be Licensed?

The action of the Buffalo Medical and Surgical Association in appointing a committee to draft a bill for presentation to the legislature, providing for the licensing of midwives, after due examination by a Board of legally qualified physicians, has been supplemented by that of the Medical Society of the County of Erie, which latter has endorsed the action of the former, adopted the draft of the bill as presented by the committee, and referred it to the legislature with the recommendation that it be passed. The provisions of the bill are, in our view, so entirely just to the midwife, so conservative of her best interests, and so far from containing onerous requirements, that we cannot believe it will receive any well-considered, or formidable opposition.

The following is a synopsis of the bill as adopted: Sec. 1 provides that on or after July 1, 1885, the County Judge of Erie county shall appoint an Examining Board of five physicians, and also provides for the filling of any vacancy. Sec. 2 designates the officers of the Board as a President, Secretary and Treasurer, and requires the adoption of rules for the conduct of the examinations. Sec. 3 requires the Board to meet on the first Tuesdays in April and October in each year, for the examination of all candidates who present themselves, who are of lawful age and of good moral character; provides, also, that ten dollars shall be the fee for the examination, which shall be applied towards defraying the expenses of the Board. Sec. 4 designates any successful candidate as a "midwife," and that she shall receive a certificate which shall entitle her to attend any case of normal labor, but provides that she shall not use instruments, attempt version, nor administer any dangerous drug, except when the attendance of a physician cannot be procured. Sec. 5 provides for the revocation of the certificate for cause. Sec. 6 prohibits the practicing of midwives after December 31, 1885, except under provisions of this bill, under a penalty of not less than fifty, nor more than one hundred dollars.

The passage of this, or some similar bill, would prove of benefit to the midwives themselves, as well as afford protection to the community against the exercise of that function by incompetent persons. While many of these women are very well-disposed persons, kind and efficient nurses, and perform their work conscientiously, yet, on the other hand, some of them are incompetent, ignorant, and unscrupulous individuals, who offer their services to whomsoever may stand in need. The former class deserve the considerate protection of the law; while the latter should be prohibited, under penalty, from the assumption of duties for which they are unfitted.

It seems almost incredible that the care of the parturient woman should be committed, in this enlightened age, to the care of persons who have not shown their competency for the trust, either by clinical experience, or preparation in the schools; and it is high time for intelligent communities to move in this matter, to the end that one of the most important services which one human being can perform for another—a service which always involves the safety of two lives—shall not be entrusted to unsafe hands. How much of woe, and suffering, and invalidism, reaching through all the after-coming years, is involved in unskillful management in the lying-in chamber, no one can compute; but it is highly probable, and this statement is made upon high authority, that the gynecologist derives the largest proportion of his patients from this source.

That a very considerable number of lacerations of the cervix, of the perinæum, and rents of the genital tract, result from the improper or indifferent care of midwives, no physician, who is at all familiar with the facts, will pretend to dispute. Cases of puerperal septicæmia from imperfect delivery of the secundines, post partum hemorrhage from the same and other causes, and a vast number of still-born children, attest the incompetency, ignorance, and wanton neglect of midwives in every large city. May other evils, due to their utter lack of proper regard for the responsibility of their position, might be cited, but enough have already been mentioned to show the importance of making some attempt to protect the lying-in chamber, be it ever so humble, from such dangers in the future.

Some very intelligent people, not a few in number, are of the opinion that the medical colleges should demand a higher standard for the doctorate degree than has heretofore been the case; others, again, assert with a cogency of force that is well-nigh convincing to even the colleges themselves, that the right to confer a license to practice medicine should be vested in a Board created by the State, wholly independent of the teaching power; while, yet again, it has lately been asserted that a diploma from a recognized university or scientific school will be required, within the next decade or two, as the *sine qua non* for entrance into a medical college. If all this preparation is demanded of the physician, before the sanctity of the lying-in room can be committed to his keeping, it certainly is not too much to ask that the untutored and non-scientific midwife shall submit herself to some scrutiny in regard to her moral character, and as to her familiarity with the simplest duties which pertain to her great responsibilities. The welfare of the community, the safety of the citizen, and the best interests of the midwife, alike appeal to the State for protection in a way which should command the thoughtful attention of the law-making power, involving as it does the tenderest and choicest solicitude of every father and mother in the commonwealth.

